# Different Effects of Total Bilirubin on 90-Day Mortality in Hospitalized Patients With Cirrhosis and Advanced Fibrosis: A Quantitative Analysis

**DOI:** 10.3389/fmed.2021.704452

**Published:** 2021-06-23

**Authors:** Liang Qiao, Wenting Tan, Xiaobo Wang, Xin Zheng, Yan Huang, Beiling Li, Zhongji Meng, Yanhang Gao, Zhiping Qian, Feng Liu, Xiaobo Lu, Jia Shang, Junping Liu, Huadong Yan, Wenyi Gu, Yan Zhang, Xiaomei Xiang, Yixin Hou, Qun Zhang, Yan Xiong, Congcong Zou, Jun Chen, Zebing Huang, Xiuhua Jiang, Sen Luo, Yuanyuan Chen, Na Gao, Chunyan Liu, Wei Yuan, Xue Mei, Jing Li, Tao Li, Rongjiong Zheng, Xinyi Zhou, Jinjun Chen, Guohong Deng, Weituo Zhang, Hai Li

**Affiliations:** ^1^Department of Gastroenterology, School of Medicine, Ren Ji Hospital, Shanghai Jiao Tong University, Shanghai, China; ^2^Key Laboratory of Gastroenterology and Hepatology, Shanghai Institute of Digestive Disease, Chinese Ministry of Health, Shanghai Jiao Tong University, Shanghai, China; ^3^Department of Infectious Diseases, Southwest Hospital, Third Military Medical University (Army Medical University), Chongqing, China; ^4^Center of Integrative Medicine, Beijing Ditan Hospital, Capital Medical University, Beijing, China; ^5^Department of Infectious Diseases, Tongji Medical College, Institute of Infection and Immunology, Union Hospital, Huazhong University of Science and Technology, Wuhan, China; ^6^Hunan Key Laboratory of Viral Hepatitis, Department of Infectious Diseases, Xiangya Hospital, Central South University, Changsha, China; ^7^Hepatology Unit, Department of Infectious Diseases, Nanfang Hospital, Southern Medical University, Guangzhou, China; ^8^Department of Infectious Disease, Taihe Hospital, Hubei University of Medicine, Shiyan, China; ^9^Department of Hepatology, The First Hospital of Jilin University, Changchun, China; ^10^Department of Liver Intensive Care Unit, Shanghai Public Health Clinical Centre, Fudan University, Shanghai, China; ^11^Tianjin Institute of Hepatology, Nankai University Second People's Hospital, Tianjin, China; ^12^Department of Infectious Diseases and Hepatology, The Second Hospital of Shandong University, Jinan, China; ^13^Infectious Disease Center, The First Affiliated Hospital of Xinjiang Medical University, Urumqi, China; ^14^Department of Infectious Diseases, Henan Provincial People's Hospital, Zhengzhou, China; ^15^Department of Infectious Diseases, Hwamei Hospital, Ningbo No. 2 Hospital, University of Chinese Academy of Sciences, Ningbo, China; ^16^Clinical Research Center, Shanghai Jiao Tong University School of Medicine, Shanghai, China

**Keywords:** liver failure, cutoff, quantitative analyse, short-term mortality, total bilirubin

## Abstract

**Introduction:** Total bilirubin (TB) is a major prognosis predictor representing liver failure in patients with acute on chronic liver failure (ACLF). However, the cutoff value of TB for liver failure and whether the same cutoff could be applied in both cirrhotic and non-cirrhotic patients remain controversial. There is a need to obtain the quantitative correlation between TB and short-term mortality *via* evidence-based methods, which is critical in establishing solid ACLF diagnostic criteria.

**Methods:** Patients hospitalized with cirrhosis or advanced fibrosis (FIB-4 > 1.45) were studied. TB and other variables were measured at baseline. The primary outcome was 90-day transplantation-free mortality. Multi-variable Cox proportional hazard model was used to present the independent risk of mortality due to TB. Generalized additive model and second derivate (acceleration) were used to plot the “TB-mortality correlation curves.” The mathematical (maximum acceleration) and clinical (adjusted 28-day transplantation-free mortality rate reaching 15%) TB cutoffs for liver failure were both calculated.

**Results:** Among the 3,532 included patients, the number of patients with cirrhosis and advanced fibrosis were 2,592 and 940, respectively, of which cumulative 90-day mortality were 16.6% (430/2592) and 7.4% (70/940), respectively. Any increase of TB was found the independent risk factor of mortality in cirrhotic patients, while only TB >12 mg/dL independently increased the risk of mortality in patients with advanced fibrosis. In cirrhotic patients, the mathematical TB cutoff for liver failure is 14.2 mg/dL, with 23.3% (605/2592) patients exceeding it, corresponding to 13.3 and 25.0% adjusted 28- and 90-day mortality rate, respectively. The clinical TB cutoff for is 18.1 mg/dL, with 18.2% (471/2592) patients exceeding it. In patients with advanced fibrosis, the mathematical TB cutoff is 12.1 mg/dL, 33.1% (311/940) patients exceeding it, corresponding to 2.9 and 8.0% adjusted 28- and 90-day mortality rate, respectively; the clinical TB cutoff was 36.0 mg/dL, 1.3% (12/940) patients above it.

**Conclusion:** This study clearly demonstrated the significantly different impact of TB on 90-day mortality in patients with cirrhosis and advanced fibrosis, proving that liver failure can be determined by TB alone in cirrhosis but not in advanced fibrosis. The proposed TB cutoffs for liver failure provides solid support for the establishment of ACLF diagnostic criteria.

## Introduction

The disease burden of acute on chronic liver failure (ACLF) is enormous. It affects 10–35% hospitalized patients with cirrhosis (1–4) and a considerable part of patients with non-cirrhotic chronic liver diseases (5, 6). ACLF is associated with sharply increased risk of short-term mortality (4, 6–10) and heavy financial costs (11, 12). The presence of organ failure is a prerequisite for the diagnosis of ACLF (4, 13, 14). In East Asia, where hepatitis B virus (HBV) infection is highly endemic (15, 16), liver failure is the most common type of organ failure (10, 17). Therefore, a clear definition of liver failure is essential in ACLF diagnosis in HBV high-endemic areas.

Though high total bilirubin (TB) has been confirmed to be strongly associated with poor liver preservation and high short-term mortality (7, 13, 18, 19), there are fierce controversies between the East and the West on the application of TB in the diagnosis of liver failure. The European Association for the Study of the Liver - Chronic Liver Failure (EASL-CLIF) defined liver failure using TB alone (TB > 12 mg/dL) (4, 20). While in Asia, liver failure was defined more frequently with a combination of TB and international normalized ratio (INR), such as the Asia-Pacific Association for the Study of the Liver (APASL) criteria (TB > 5 mg/dL and INR > 1.5) (21) and the Chinese Group on the Study of Severe Hepatitis B (COSSH) criteria (TB > 12 mg/dL and INR > 1.5) (6). The inequality was mainly due to whether patients with non-cirrhotic chronic liver diseases included in the respective study (2, 4, 6, 14, 22).

The key to resolving these controversies on the definition of liver failure is to clarify whether the liver failure of cirrhosis and non-cirrhotic chronic liver diseases (mainly advanced fibrosis) can be diagnosed by the same criteria. Given the absence of studies evaluating this specific issue, we aimed to describe the quantitative correlation between the baseline TB level and the 90-day mortality among hospitalized patients with cirrhosis and advanced fibrosis, respectively. The detailed and intuitive presentation of this correlation is crucial to define liver failure and establish the solid ACLF diagnostic criteria.

## Materials and Methods

### Study Design, Setting, and Oversight

We combined the data from two prospective observational cohorts of the Chinese AcuTe on CHronic LIver FailurE (CATCH-LIFE) study: the CATCH-LIFE investigation cohort (NCT02457637) involving 14 hospitals throughout China, enrollment occurred from January 2015 to December 2016; and the CATCH-LIFE validation cohort (NCT03641872), involving 13 hospitals throughout China, enrollment occurred from September 2018 to March 2019. All participants were followed-up for at least 90 days. The study protocols and baseline characteristics have been described in detail elsewhere (23, 24). The medical ethics boards of Shanghai Renji Hospital (the lead center of the CATCH-LIFE study), approved the study [ethics code: (2014)148 k and (2016)142 k]. Written informed consent were obtained from every participant or his/her legal surrogates before enrollment.

### Study Population

Eligible patients were required with cirrhosis or advanced fibrosis hospitalized with at least one of the following criteria: (1) with acute decompensations (ADs), including overt ascites (25), gastrointestinal bleeding, hepatic encephalopathy (HE) (26), bacterial infection, or jaundice (TB > 5 mg/dL)] within 1 month before enrollment; (2) with acute liver injury, including alanine aminotransferase (ALT), or aspartate aminotransferase (AST) >3 upper limitation of normal level or total bilirubin (TB) >2 upper limitation of normal level within recent 1 week before enrollment.

Patients with cirrhosis and advanced fibrosis were divided into two groups for comparison. Cirrhosis was diagnosed according to the signs of dysmorphia and relation to liver fibrosis and portal hypertension in the imaging examination (computerized tomography, magnetic resonance imaging or abdominal ultrasound) after enrolment. The cirrhotic patients with a history of decompensation at least 1 month ago are defined to have decompensated cirrhosis, and other cirrhotic patients without any history of decompensation (including the patients hospitalized due to the first episode of decompensation events) are defined to have compensatory cirrhosis. Non-cirrhotic patients with a fibrosis-4 (FIB-4) score (27) < 1.45 were excluded to rule-out those who with merely mild or no fibrosis.

### Exposure and Outcomes

The primary exposure was TB level (mg/dL), which was measured at admission. Other clinical data were measured and collected at admission as well, including demographics (age, sex), etiology of underlying liver diseases (HBV related, alcohol related or others), complications (type of ADs), laboratory findings and severity scores [the model of end-stage liver diseases [MELD] (18), MELD-Sodium (28), and Child-Turcotte-Pugh (29)].

The primary outcome was the 90-day mortality (transplantation-free). The patients who received liver transplantation (LT) within 90 days were excluded to present the correlation between baseline TB level and patients' natural 90-day prognosis.

### Statistical Analysis

Continuous variables were summarized by mean and standard deviation (SD) or median or inter-quartile range (IQR) based on their distribution. Categorical variables were summarized by frequency and proportion [with 95% confidence interval (CI)].

The correlation between TB and 90-day mortality were analyzed using a multivariable Cox proportional hazard (COXPH) model, adjusting for important risk factors and potential confounders, including age, sex, etiologies of underlying chronic liver diseases, presence or absence of overt ascites, gastrointestinal bleeding and bacterial infection, the grades of HE, the level of INR, creatinine, ALT, and serum sodium. The risk of 90-day mortality was expressed as a continuous variable with hazard ratios (HRs) calculated per mg/dL increment of TB, and as a categorical variable based on different range of TB levels as following: <2, 2–5, 5–8, 8–12, 12–16, 16–20, and >20 mg/dL; the patients with lowest TB (<2 mg/dL) were taken as the reference.

The non-lineal relationship in 90-day mortality over the range of TB level (0–50 mg/dL) was plotted as a “TB-mortality correlation curve.” The estimated mortality corresponding to the TB values in the curves was adjusted for above listed confounding factors by the generalized additive model (GAM) (30) to present the independent impact of TB on mortality. Spline (31) was taken as connection function in GAM and the smoothing parameters were chosen to optimize the Akaike Information Criterion. Second derivative (acceleration) (32) of TB to mortality was used to describe the non-lineal relationship.

Finally, based on the multivariable adjusted TB-mortality correlation curves, the TB values that correspond to the inflection points (maximum of acceleration) of the curves would be taken as the mathematical cutoff. Additionally, the TB value that correspond to adjusted 28-day transplantation-free mortality reaching 15% (the definition of EASL-CLIF) (4) would be calculated as well and taken as clinical cutoff for liver failure.

The analysis above mentioned will be performed in patients with cirrhosis and advanced fibrosis, respectively to compare the characteristics of them. The software environment R 4.0.0 and MATLAB 2016b were used for the analysis. The R package *gam* was used for the GAM fitting and the MATLAB tool box *curve fitting* was used for curve fitting and second derivative calculation. All tests were 2-sided with α = 0.05.

## Results

### Enrollment and Baseline Characteristics

**Figure 1:**
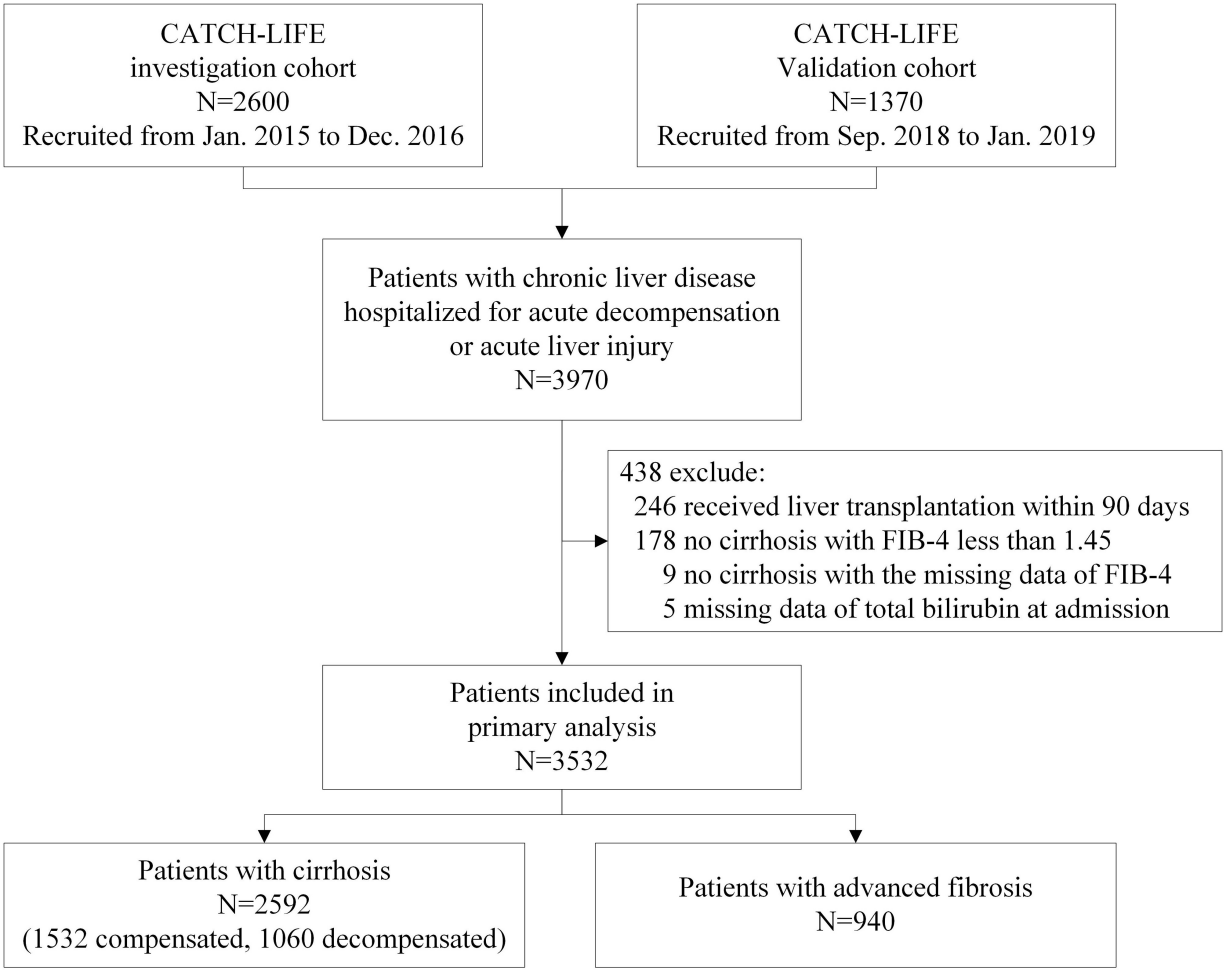
Flowchart of the study.

**Figure 2:**
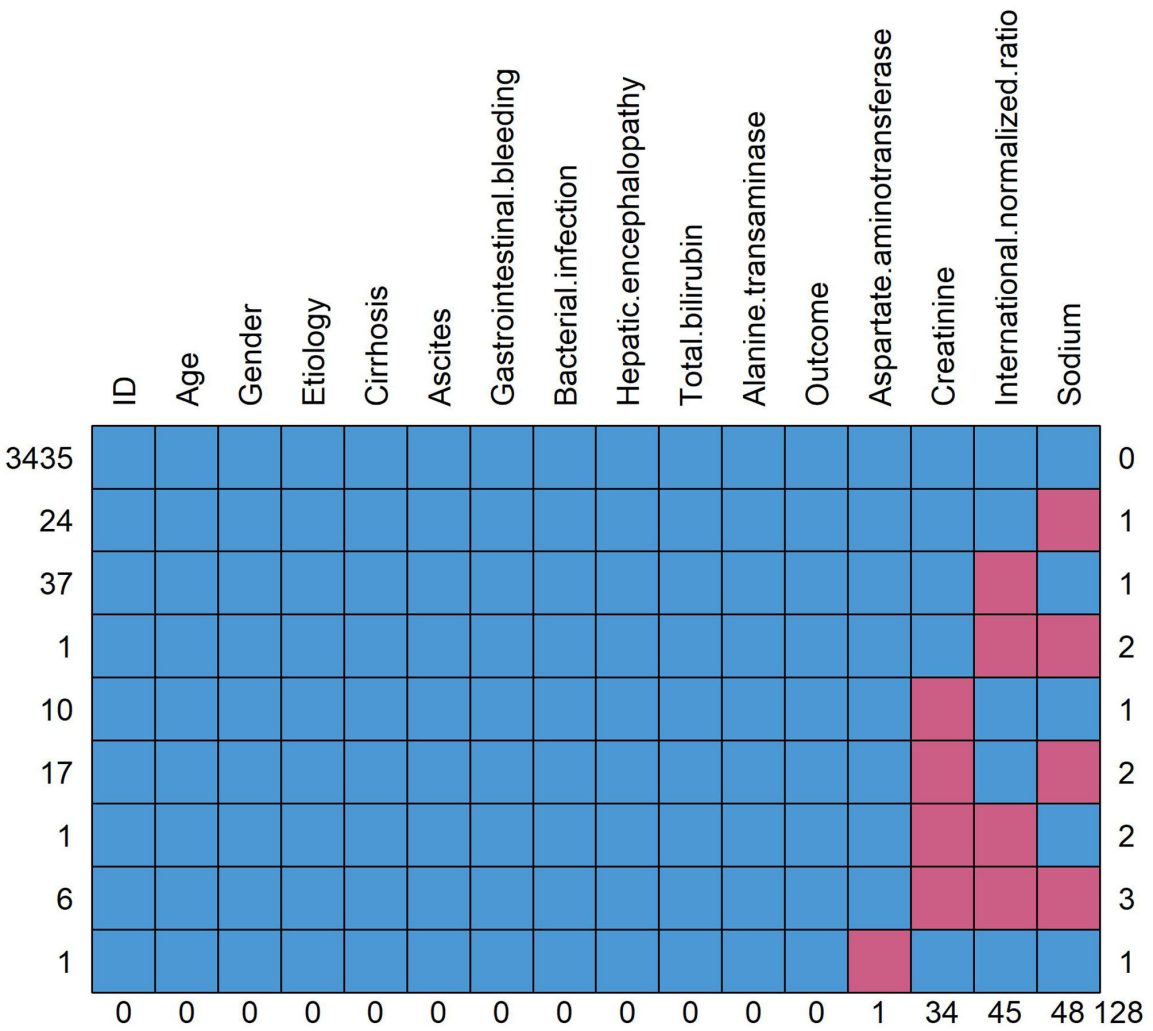
Pattern of missing data. The red square means data missing and the blue square means data complete. The figure shows the overall number of missing data for each variable and the distribution of different missing types. Taking the “sodium” in the rightmost column as an example, a total of 48 patients have missing data of sodium, of which 24 patients (line 2) have missing data of sodium alone, 1 patient (line 4) has missing data of international standardized ratio and sodium, 17 patients (line 6) have missing data of creatinine and sodium and 6 patients (line 8) have missing data of creatinine, international normalized ratio and sodium.

A total of 3,532 patients were included in this analysis (Figure 1). Among them, 2,592 (73.4%) patients had cirrhosis (including 1,532 compensated and 1,060 decompensated), and 940 (26.6%) patients had advanced fibrosis. Patients underwent LT within 90 days (*n* = 246), with non-cirrhosis chronic liver diseases and a FIB-4 score < 1.45 (*n* = 178), or with missing TB values (*n* = 5), or FIB-4 score (*n* = 9) were excluded. All patients included finished the 90-day follow-up. The cumulative 90-day transplantation-free mortality of the patients with cirrhosis and advanced fibrosis were 16.6% (430/2592) (Table 1) and 7.4% (70/940), respectively (Table 2). Demographic, clinical characteristics and 90-day transplantation-free mortality of patients with cirrhosis and advanced fibrosis are presented in Tables 1, 2, respectively according to different TB level. The pattern of missing data can be found in Figure 2.

**Table 1:** Comparison of baseline characteristics of patients with cirrhosis based on different level of total bilirubin (mg/dL) at admission.

**Variable**	**0<TB≤2**	**2<TB≤5**	**5<TB≤8**	**8<TB≤12**	**12<TB≤16**	**16<TB≤20**	**20<TB**
	***N* = 839**	***N* = 601**	***N* = 215**	***N* = 220**	**N = 172**	***N* = 139**	***N* = 406**
**Demographics**							
Age, mean (SD)	54.4 (11.4)	52.2 (11.0)	51.1 (11.4)	50.8 (11.4)	49.0 (11.1)	48.1 (10.4)	48.3 (11.3)
Gender, No. (%)	553 (65.9)	439 (73.0)	149 (69.3)	161 (73.2)	124 (72.1)	112 (80.6)	351 (86.5)
**Etiology, No. (%)**							
HBV	486 (57.9)	366 (60.9)	131 (60.9)	123 (55.9)	118 (68.6)	96 (69.1)	307 (75.6)
Alcoholic	101 (12.0)	78 (13.0)	29 (13.5)	36 (16.4)	16 (9.3)	12 (8.6)	27 (6.7)
Others	252 (30.0)	157 (26.1)	55 (25.6)	61 (27.7)	38 (22.1)	31 (22.3)	72 (17.7)
**Complications, No. (%)**							
Ascites	481 (57.3)	369 (61.4)	131 (60.9)	138 (62.7)	104 (60.5)	98 (70.5)	285 (70.2)
Gastrointestinal bleeding	342 (40.8)	99 (16.5)	26 (12.1)	13 (5.9)	10 (5.8)	4 (2.9)	20 (4.9)
Bacterial infection	134 (16.0)	128 (21.3)	58 (27.0)	75 (34.1)	58 (33.7)	44 (31.7)	196 (48.3)
Hepatic encephalopathy							
Not overt	805 (95.9)	564 (93.8)	197 (91.6)	205 (93.2)	161 (93.6)	136 (97.8)	350 (86.2)
Grade 2	25 (3.0)	26 (4.3)	10 (4.7)	9 (4.1)	10 (5.8)	2 (1.4)	37 (9.1)
Grade 3	7 (0.8)	6 (1.0)	8 (3.7)	4 (1.8)	0 (0.0)	1 (0.7)	14 (3.4)
Grade 4	2 (0.2)	5 (0.8)	0 (0.0)	2 (0.9)	1 (0.6)	0 (0.0)	5 (1.2)
**Laboratory results, median (IQR)**							
Hemoglobin, g/L	97.0 (75.0, 118.0)	113.0 (95.0, 128.0)	105.0 (88.5, 122.0)	110.2 (89.0, 131.9)	115.5 (96.8, 129.0)	118.0 (107.2, 132.0)	119.0 (103.0, 133.4)
White blood cell, 10^9^/L	3.9 (2.6, 5.5)	4.1 (3.0, 6.0)	4.8 (3.4, 6.4)	5.3 (3.7, 7.0)	5.7 (4.1, 7.8)	6.1 (4.7, 8.1)	7.1 (5.0, 10.1)
Platelet, 10^9^/L	76.0 (51.0, 122.0)	64.0 (42.9, 98.0)	71.0 (46.0, 106.5)	76.0 (48.9, 118.5)	80.5 (54.0, 123.2)	92.0 (63.5, 128.0)	85.0 (58.0, 121.0)
International normalized ratio	1.3 (1.1, 1.4)	1.5 (1.3, 1.6)	1.6 (1.4, 1.9)	1.7 (1.4, 2.1)	1.9 (1.5, 2.4)	1.9 (1.5, 2.4)	2.1 (1.6, 2.7)
Creatinine, mg/dL	0.8 (0.7, 1.0)	0.8 (0.6, 0.9)	0.7 (0.6, 0.9)	0.7 (0.6, 1.0)	0.8 (0.6, 1.0)	0.8 (0.6, 1.0)	0.9 (0.7, 1.2)
Albumin, g/L	31.9 (27.6, 36.0)	29.2 (25.2, 33.1)	28.3 (24.5, 34.0)	29.7 (26.3, 33.4)	29.8 (26.6, 33.0)	29.6 (26.2, 33.0)	31.0 (27.9, 34.0)
Alanine transaminase, IU/L	28.0 (17.7, 51.5)	45.3 (27.0, 89.6)	58.1 (31.0, 151.4)	100.6 (33.2, 391.9)	102.4 (45.5, 372.6)	206.3 (85.3, 566.6)	159.3 (61.5, 385.3)
Aspartate transaminase, IU/L	37.0 (24.6, 62.9)	65.0 (42.0, 116.6)	92.3 (51.0, 179.0)	126.3 (62.9, 330.0)	145.4 (77.3, 291.5)	191.8 (110.1, 429.3)	177.9 (100.8, 332.8)
Sodium, mmol/L	139.7 (137.0, 141.8)	138.6 (135.8, 141.0)	137.4 (134.9, 140.0)	137.3 (133.4, 139.5)	137.3 (133.3, 139.8)	136.9 (133.9, 138.9)	135.0 (131.0, 138.0)
**Score**, **mean (SD)**							
MELD	9.7 (4.0)	14.4 (3.9)	18.2 (5.0)	20.8 (4.6)	23.0 (4.8)	25.1 (5.1)	28.6 (5.7)
MELD-Na	10.5 (5.1)	15.7 (5.1)	19.6 (6.6)	22.6 (4.8)	24.5 (5.5)	26.6 (4.9)	30.0 (5.3)
CTP	7.3 (1.4)	9.4 (1.5)	10.2 (1.6)	10.3 (1.7)	10.5 (1.8)	10.6 (1.7)	10.8 (1.7)
**Transplantation-free mortality**							
28-day, No. (%)	17 (2.0)	26 (4.3)	14 (6.5)	19 (8.6)	22 (12.8)	26 (18.7)	117 (28.8)
90-day, No. (%)	35 (4.2)	51 (8.5)	25 (11.6)	36 (16.4)	38 (22.1)	44 (31.7)	201 (49.5)

*SD, standard deviation; HBV, hepatitis B virus; IQR, interquartile range; MELD, the model of end-stage liver disease; MELD-Sodium, the model of end-stage liver disease with sodium; CTP, Child-Turcotte-Pugh*.

**Table 2:** Comparison of baseline characteristics of patients with advanced fibrosis based on different level of total bilirubin (mg/dL) at admission.

**Variable**	**0<TB≤2**	**2<TB≤5**	**5<TB≤8**	**8<TB≤12**	**12<TB≤16**	**16<TB≤20**	**20<TB**
	***N* = 239**	***N* = 188**	***N* = 96**	***N* = 104**	***N* = 103**	***N* = 74**	***N* = 136**
**Demographics**							
Age, mean (SD)	44.8 (12.7)	42.1 (11.3)	41.4 (12.5)	43.8 (12.0)	42.5 (11.2)	43.8 (11.6)	43.3 (12.5)
Gender, No. (%)	159 (66.5)	146 (77.7)	76 (79.2)	72 (69.2)	79 (76.7)	60 (81.1)	114 (83.8)
**Etiology**, **No. (%)**							
HBV	160 (66.9)	133 (70.7)	71 (74.0)	77 (74.0)	86 (83.5)	47 (63.5)	113 (83.1)
Alcoholic	9 (3.8)	5 (2.7)	3 (3.1)	2 (1.9)	2 (1.9)	2 (2.7)	2 (1.5)
Others	70 (29.3)	50 (26.6)	22 (22.9)	25 (24.0)	15 (14.6)	25 (33.8)	21 (15.4)
**Complications**, **No. (%)**							
Ascites	6 (2.5)	6 (3.2)	9 (9.4)	15 (14.4)	19 (18.4)	14 (18.9)	51 (37.5)
Bacterial infection	10 (4.2)	8 (4.3)	7 (7.3)	9 (8.7)	16 (15.5)	16 (21.6)	41 (30.1)
Hepatic encephalopathy							
Not overt	239 (100.0)	188 (100.0)	96 (100.0)	104 (100.0)	99 (96.1)	72 (97.3)	122 (89.7)
Grade 2	0 (0.0)	0 (0.0)	0 (0.0)	0 (0.0)	2 (1.9)	0 (0.0)	8 (5.9)
Grade 3	0 (0.0)	0 (0.0)	0 (0.0)	0 (0.0)	1 (1.0)	2 (2.7)	4 (2.9)
Grade 4	0 (0.0)	0 (0.0)	0 (0.0)	0 (0.0)	1 (1.0)	0 (0.0)	2 (1.5)
**Laboratory results**, **median (IQR)**							
Hemoglobin, g/L	141.0 (127.0, 152.0)	138.0 (128.8, 153.0)	136.0 (121.5, 145.2)	136.0 (117.2, 147.0)	136.0 (124.5, 151.0)	130.0 (114.8, 142.0)	128.0 (115.0, 144.0)
White blood cell, 10^9^/L	4.6 (4.0, 5.4)	4.7 (3.9, 5.9)	5.2 (4.1, 6.3)	5.2 (4.2, 6.6)	6.2 (5.0, 7.6)	6.6 (5.2, 7.9)	7.2 (5.7, 9.8)
Platelet, 10^9^/L	147.0 (113.0, 187.2)	139.5 (103.2, 177.5)	128.0 (102.0, 168.5)	136.5 (94.8, 171.2)	132.0 (98.0, 160.0)	129.0 (98.0, 173.5)	122.0 (90.2, 166.0)
International normalized ratio	1.1 (1.0, 1.2)	1.2 (1.0, 1.3)	1.3 (1.1, 1.6)	1.4 (1.1, 1.7)	1.6 (1.3, 2.1)	1.5 (1.2, 2.0)	1.8 (1.5, 2.5)
Creatinine, mg/dL	0.7 (0.6, 0.9)	0.8 (0.7, 0.9)	0.7 (0.6, 0.8)	0.7 (0.6, 0.9)	0.7 (0.6, 0.8)	0.8 (0.7, 0.9)	0.8 (0.6, 1.0)
Albumin, g/L	40.1 (36.8, 43.5)	38.3 (34.2, 42.0)	35.9 (33.0, 39.5)	34.5 (31.8, 37.9)	33.2 (30.0, 37.4)	32.7 (29.3, 36.2)	32.2 (29.9, 35.6)
Alanine transaminase, IU/L	408.0 (184.0, 751.5)	790.0 (383.5, 1137.0)	832.0 (310.5, 1360.0)	653.0 (301.1, 1331.2)	639.4 (273.2, 1275.5)	509.9 (147.2, 952.0)	302.4 (145.0, 818.1)
Aspartate transaminase, IU/L	248.0 (144.0, 431.2)	428.9 (220.0, 750.2)	535.5 (223.1, 1007.2)	508.4 (267.7, 908.2)	433.0 (197.0, 949.7)	284.6 (153.7, 680.0)	207.1 (144.8, 571.2)
Sodium, mmol/L	140.4 (138.0, 142.0)	140.0 (138.0, 141.4)	139.0 (136.5, 140.8)	139.0 (136.2, 140.8)	138.0 (136.0, 140.0)	137.0 (135.0, 139.0)	137.2 (135.0, 140.0)
**Score**, **mean (SD)**							
MELD	8.2 (2.8)	12.4 (3.3)	16.6 (3.0)	19.1 (3.9)	21.5 (4.2)	23.1 (4.8)	26.8 (5.7)
MELD-Na	8.1 (4.0)	12.7 (3.9)	17.5 (3.5)	20.1 (4.0)	22.4 (4.3)	24.3 (4.8)	27.7 (5.6)
CTP	5.3 (0.8)	6.9 (1.0)	7.8 (1.0)	8.3 (1.3)	8.7 (1.6)	8.9 (1.7)	9.6 (1.9)
**Transplantation-free mortality**							
28-day, No. (%)	0 (0.0)	1 (0.5)	0 (0.0)	1 (1.0)	7 (6.8)	4 (5.4)	27 (19.9)
90-day, No. (%)	3 (1.3)	2 (1.1)	2 (2.1)	5 (4.8)	10 (9.7)	11 (14.9)	37 (27.2)

*SD, standard deviation; HBV, hepatitis B virus; IQR, interquartile range; MELD, the model of end-stage liver disease; MELD-Sodium, the model of end-stage liver disease with sodium; CTP, Child-Turcotte-Pugh*.

### Any Increase of TB Independently Increased the Mortality Risk in Cirrhosis

In patients with cirrhosis, any increase in TB, either expressed as a continuous variable or categorical variable, was the risk factor of 90-day mortality in all univariable and multivariable-adjusted analysis. The full-adjusted HR was 1.060 (95% CI, 1.051–1.069) per 1 mg/dL increase of TB (Table 3). Categorical-variable analysis showed that compared with the cirrhotic patients with normal TB (<2 mg/dL), the HRs were significantly higher in patients within any other categories of elevated TB (*p*<0.001 for all). Moreover, the HRs were positively correlated with the TB level of patients *p* (for trend = 0.002).

**Table 3:** The unadjusted and adjusted hazard ratios of 90-day transplantation-free mortality due to total bilirubin in patients with cirrhosis.

**Total bilirubin** ** (mg/dL)**		**Number of patients**	**Number of death (%)**	**Hazard ratio of transplantation-free mortality (95% CI)**, ***p*****-value**	
				**Unadjusted**	Adjusted^*^
As a continuous variable		2,592	430 (15.3)	1.077 (1.070–1.084), <0.001	1.060 (1.051–1.069), <0.001
As a categorical variable	0–2	839	35 (3.9)	1 (Reference)	1 (Reference)
	2–5	601	51 (8)	2.081 (1.354–3.200), 0.001	2.120 (1.364–3.295), 0.001
	5–8	215	25 (10.9)	2.915 (1.745–4.870), <0.001	2.520 (1.467–4.331), 0.001
	8–12	220	36 (15.7)	4.170 (2.619–6.640), <0.001	3.527 (2.154–5.776), <0.001
	12–16	172	38 (20.5)	5.858 (3.701–9.273), <0.001	4.808 (2.934–7.878), <0.001
	16–20	139	44 (28.8)	8.988 (5.765–14.012), <0.001	7.927 (4.861–12.928), <0.001
	Over 20	406	201 (42.8)	16.171 (11.289–23.166), <0.001	10.422 (6.874–15.802), <0.001
	*P*-value for trend			0.004	<0.001

* *Adjusted for age, gender, etiology, hepatic encephalopathy grade, infection, ascites, gastrointestinal bleeding, international normalized ratio, creatinine, serum sodium and alanine transaminase*.

### The Mortality Risk Increased only if TB Is >12 mg/dL in Advanced Fibrosis

In patients with advanced fibrosis, though as a continuous variable, higher TB was also independently associated with 90-day mortality overall [adjusted HR of 1.080 per 1 mg/dL increase of TB, (95% CI, 1.049–1.112)]. Interestingly, categorical-variable analysis demonstrated that these effects were primarily driven by TB over 12 mg/dL (Table 4), any TB categories <12 mg/dL was not the independent risk factor of mortality. Besides the qualitative *p*-values, it can be found that compared to the HRs of the patients with TB of 2–5 mg/dL [0.802 (95% CI, 0.130–4.956), *p* = 0.812], 5–8 mg/dL [1.443 (95% CI, 0.237–8.803), *p* = 0.691], and 8–12 mg/dL [1.866 (95% CI, 0.401–8.681), *p* = 0.426], the HR in patients with TB of 12–16 mg/dL markedly elevated to 4.186 [(95% CI 1.070–16.371), *p* = 0.040], suggesting a threshold effect within the range.

**Table 4:** The unadjusted and adjusted hazard ratios of 90-day transplantation-free mortality due to total bilirubin in patients with advanced fibrosis.

**Total bilirubin** ** (mg/dL)**		**Number of patients**	**Number of death (%)**	**Hazard ratio of transplantation-free mortality (95% CI)**, ***p*****-value**	
				**Unadjusted**	Adjusted^*^
As a continuous variable		940	70 (7.3)	1.109 (1.087–1.131), <0.001	1.080 (1.049–1.112), <0.001
As a categorical variable	0–2	239	3 (1.3)	1 (Reference)	1 (Reference)
	2–5	188	2 (1.1)	0.845 (0.141–5.056), 0.854	0.802 (0.130–4.956), 0.812
	5–8	96	2 (2.1)	1.653 (0.276–9.895), 0.582	1.443 (0.237–8.803), 0.691
	8–12	104	5 (4.7)	3.887 (0.929–16.267), 0.063	1.866 (0.401–8.681), 0.426
	12–16	103	10 (9.5)	8.131 (2.238–29.545), 0.001	4.186 (1.070–16.371), 0.040
	16–20	74	11 (14.5)	12.606 (3.517–45.188), <0.001	5.829 (1.476–23.026), 0.012
	Over 20	136	37 (25.9)	25.624 (7.899–83.118), <0.001	8.455 (2.358–30.312), 0.001
	*P*-value for trend			0.008	0.002

* *Adjusted for age, gender, etiology, hepatic encephalopathy grade, infection, ascites, international normalized ratio, creatinine, serum sodium, and alanine transaminase*.

### Liver Failure Can Be Defined by TB Alone in Cirrhosis

Through the GAM and spline, we intuitively plotted the relationship between TB and multivariable-adjusted 90-day transplantation-free mortality. Both the TB-mortality correlation curves of cirrhosis and advanced fibrosis were monotonically increasing. However, the rate of change of 90-day transplantation-free mortality per mg/dL of TB was not a constant, implying the existence of non-lineal effects.

**Figure 3:**
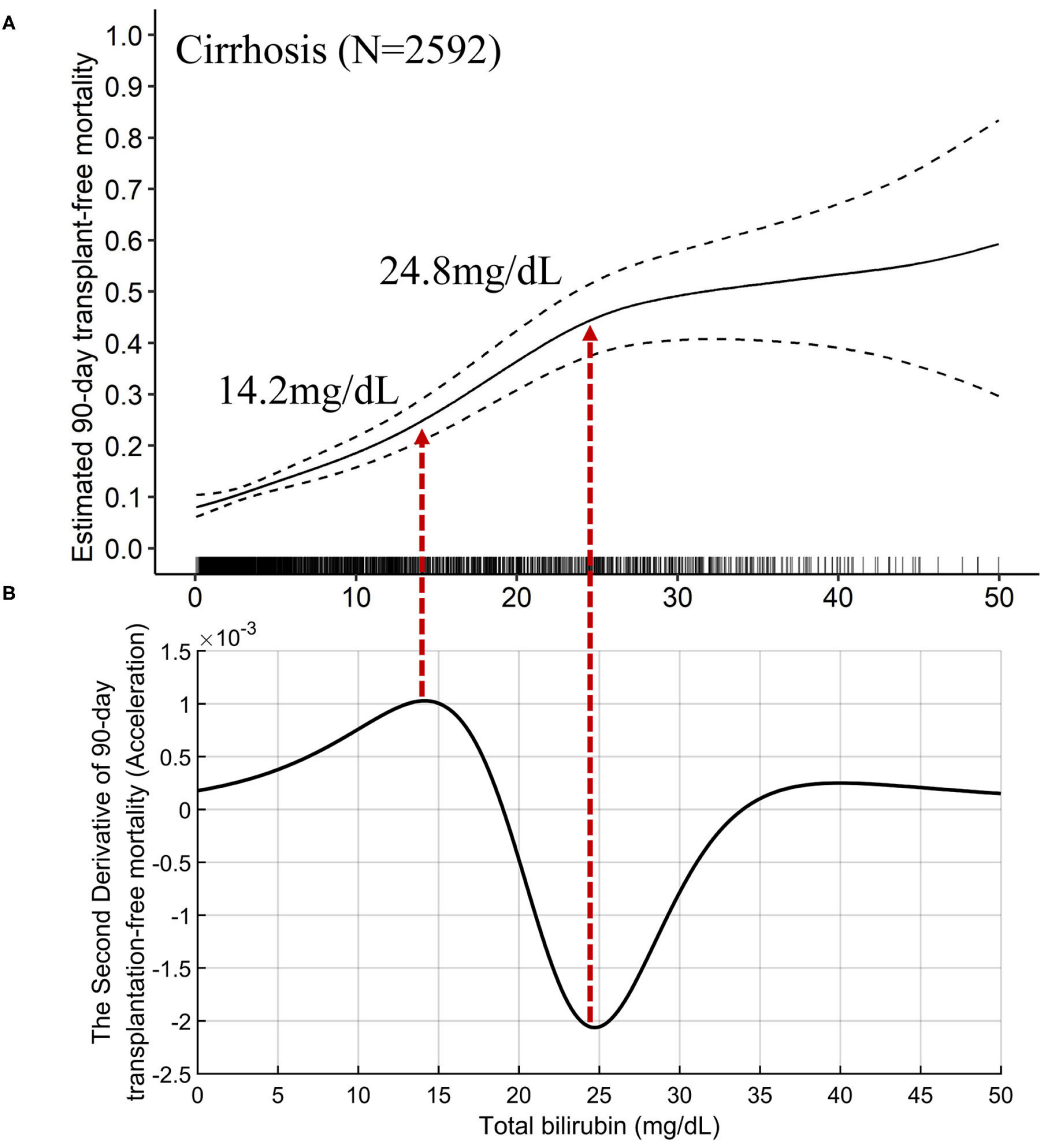
The TB-mortality (90-day) correlation curve of cirrhosis and the corresponding second derivative (acceleration) curve. **(A)** the TB-mortality correlation curve of cirrhosis; **(B)** the second derivative (acceleration) of TB to mortality. TB, total bilirubin.

The TB-mortality correlation curve of cirrhosis (Figure 3A) was S-shaped and can be roughly divided into three parts by two inflection points (TB of 14.2 and 24.8 mg/dL). The two points, respectively correspond to the maximum and minimum values (peak and valley in Figure 3B of the second derivative (acceleration) of the mortality relative to the change in TB. To be specific, when TB was between 0 and 14.2 mg/dL, the mortality acceleratingly increases with TB. When TB was between 14.2 and 24.8 mg/dL, the absolute mortality brought by per mg/dL increase of TB is the largest. When TB exceeds 24.8 mg/dL, the increase of mortality slows down, showing a saturation effect.

**Figure 4:**
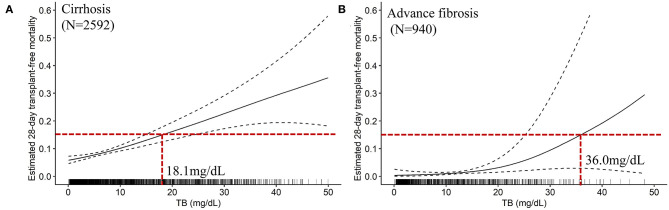
The TB-mortality (28-day) correlation curves and the clinical cutoffs of cirrhosis and advanced fibrosis. **(A)** the TB-mortality correlation curve (28-day) of cirrhosis; **(B)** the TB-mortality correlation curve (28-day) of advanced fibrosis. TB, total bilirubin.

The mathematical TB cutoff was 14.2 mg/dL, with 23.3% (605/2592) patients exceeding the cutoff. The corresponding adjusted 28- and 90-day transplantation-free mortality were 13.3 and 25.0%, respectively. Moreover, the clinical TB cutoff was 18.1 mg/dL Figure 4A, with 18.2% (471/2592) patients exceeding the cutoff. The corresponding adjusted 90-day transplantation-free mortality was 32.7%. If take the TB of 24.8 mg/dL (the valley in Figure 3B) as a cutoff, 10.3% (268/2592) patients have TB higher than the cutoff, corresponding to 19.2 and 44.8% of adjusted 28- and 90-day transplantation-free mortality, respectively.

**Figure 5:**
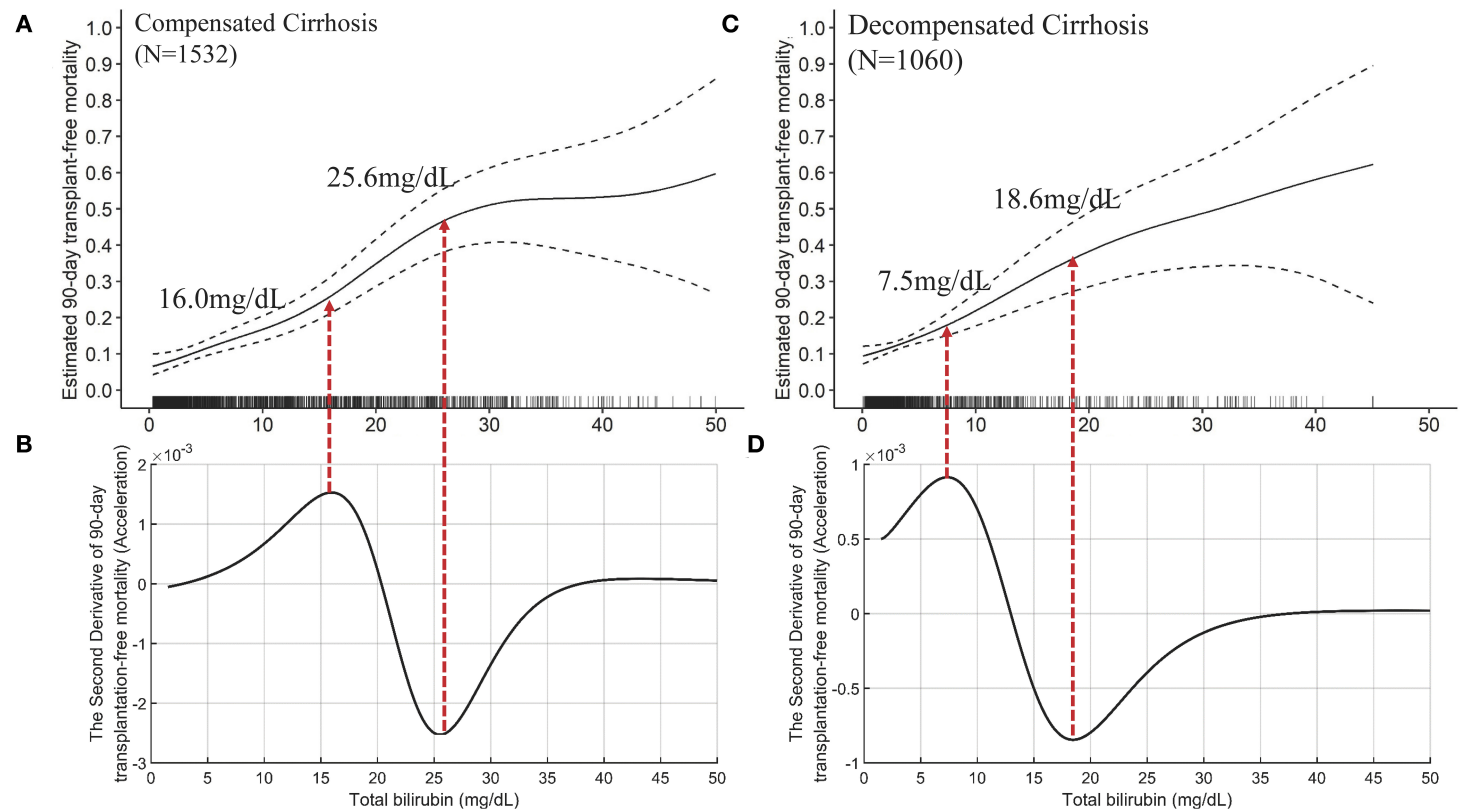
The TB-mortality (90-day) correlation curves of compensated cirrhosis and decompensated cirrhosis and their corresponding second derivative (acceleration) curves. **(A)** the TB-mortality correlation curve of compensated cirrhosis; **(B)** the second derivative (acceleration) of TB to mortality *(compensated cirrhosis); **(C)** the TB-mortality correlation curve of decompensated cirrhosis; **(D)** the second derivative (acceleration) of TB to mortality (decompensated cirrhosis). TB, total bilirubin.

We furtherly plotted the TB-mortality correlation curves of patients with compensated cirrhosis (Figures 5A,B) and decompensated cirrhosis (Figures 5C,D). The trends of the two curves were similar, and both have two inflection points. Compared with the curve of compensated cirrhosis, the curve of decompensated cirrhosis is closer to linearity, and the TB value corresponding to the two inflection points were smaller (7.5 vs. 16.0 mg/dL, and 18.6 vs. 26.0 mg/dL).

### Liver Failure Is Insufficient to Be Defined by TB Alone in Advanced Fibrosis

**Figure 6:**
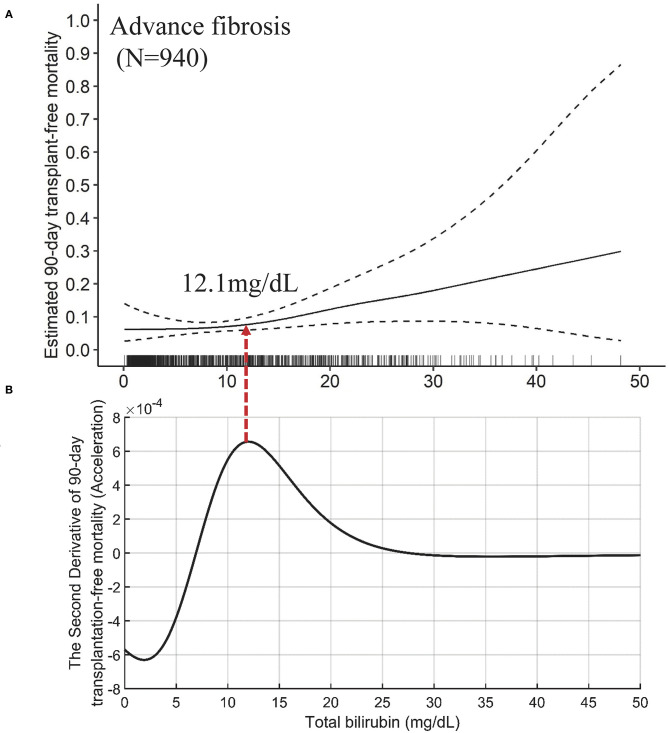
The TB-mortality (90-day) correlation curve of advanced fibrosis and the corresponding second derivative (acceleration) curve. **(A)** the TB-mortality correlation curve of advanced fibrosis; **(B)** the second derivative (acceleration) curve of TB to mortality. TB, total bilirubin.

**Figure 7:**
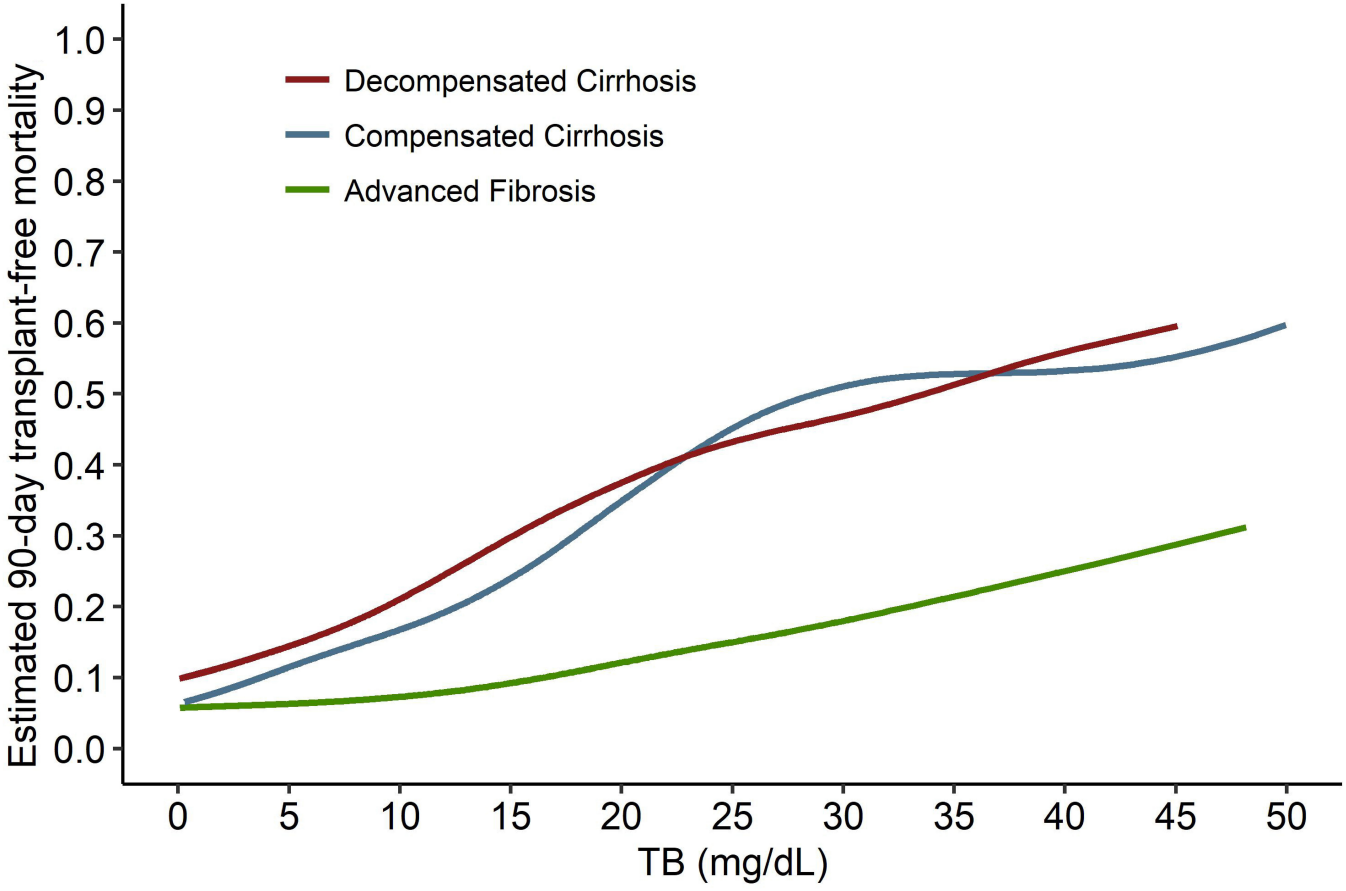
The comparison of the TB-mortality curves between decompensated cirrhosis, compensated cirrhosis, and advanced fibrosis. TB, total bilirubin.

The independent effects of TB on mortality in patients with advanced fibrosis (Figure 6A) are significantly different from that in cirrhotic patients, either compensated or decompensated (Figure 7). Compared with the TB-mortality correlation curve of cirrhosis, besides the lower overall mortality, there is only one inflection point (the peak in Figure 6B, 12.1 mg/dL) in the TB-mortality correlation curve of advanced fibrosis, dividing the curve into two parts. The TB-mortality correlation curve is almost horizontal when TB is below 12.1 mg/dL. While with TB exceeding 12.1 mg/dL, mortality rate is positively and linearly related to TB. It was consistent with the findings from the COXPH model presented in Table 4.

The mathematical TB cutoff was 12.1 mg/dL, with 33.1% (311/940) patients with advanced fibrosis exceeding the cutoff, corresponding to 2.9 and 8.0% adjusted 28- and 90-day transplantation-free mortality, respectively. The clinical TB cutoff was calculated to be 36.0 mg/dL (Figure 4B). With this cutoff, only 1.3% (12/940) patients could be stratified with a higher TB, among which 22.1% died during 90 days (Table 5).

**Table 5:** The multivariable adjusted 28-day and 90-day mortality based on the calculated the mathematical and clinical TB cutoffs for liver failure.

	**Meaning of the TB cutoff**	**Value of the cutoff**	**Percentage of patients with TB exceeding the cutoff**	**Corresponding adjusted transplantation-free mortality**	
				**28 days**	**90 days**
Cirrhosis	Peak of acceleration curve (mathematical cutoff)	14.2 mg/dL	23.3% (605/2592)	13.3%	25.0%
	Valley of acceleration curve	24.8 mg/dL	10.3% (268/2592)	19.1%	44.8%
	Reaching 15% 28-day transplantation-free mortality (clinical cutoff)	18.1 mg/dL	18.2% (471/2592)	15.0%	32.7%
Advanced fibrosis	Peak of acceleration curve (mathematical cutoff)	12.1 mg/dL	33.1% (311/940)	2.9%	8.0%
	Reaching 15% 28-day transplantation-free mortality (clinical cutoff)	36.0 mg/dL	1.3% (12/940)	15.0%	22.1%

*TB, total bilirubin*.

## Discussion

The present prospective study with large sample size first time established the respective TB cutoffs for liver failure in cirrhosis and advanced fibrosis *via* calculation. Specifically, any increase of TB independently increases the risk of 90-day transplantation free mortality in patients with cirrhosis. While, for patients with advanced fibrosis, only TB >12 mg/dL increased the risk. More importantly, based on the sufficient data of large prospective cohorts and rigorous analysis, we intuitively demonstrated the different effects of TB on the mortality in patients with cirrhosis and advanced fibrosis, and calculated their mathematical and clinical cutoffs for liver failure, respectively. The findings from this study offer an essential component of evidence-based ACLF diagnostic criteria in HBV high-endemic areas.

Western researchers represented by EASL-CLIF believe that the three major conditions of ACLF are cirrhosis, ADs and organ failure (4). They did not think that ACLF will present in non-cirrhotic patients. In contrast, the Eastern researchers represented by APASL emphasize the “reversibility of ACLF” and exclude decompensated cirrhosis out of ACLF. To achieve a universally acceptable definition, the type of ACLF were suggested to be divided into three categories depending on underlying chronic liver diseases: type A (non-cirrhotic ACLF), type B (cirrhotic ACLF), and type C (decompensated cirrhotic ACLF) (13, 33, 34). However, these subjective concepts are not appropriate to serve as solid evidence.

A clear definition of liver failure has fundamental significance for the diagnosis of ACLF. From this perspective view, our findings were obtained from quantitative analysis of the data from large cohort studies, which provides the possibility to stop this controversy. At first, we revealed the different effects of TB on mortality in patients with cirrhosis and advanced fibrosis, respectively, and proved that liver failure can be diagnosed by TB alone in cirrhosis but not in advanced fibrosis. Therefore, we demonstrated our hypothesis that the controversy between the East and the West on how to use TB to diagnose liver failure was caused by differences of the study population. Then, in the comparison of the TB-mortality correlation curves of different underlying chronic liver diseases, we found that the trend of the curve of compensated cirrhosis was similar to that of decompensated cirrhosis and quite different from that of advanced fibrosis. We observed the saturation effects in both the two curves of cirrhosis, which didn't present in the curve of advanced fibrosis. On the other hand, when TB increases from 0 to 12 mg/dL, the curves of cirrhosis rises simultaneously while the curve of advanced fibrosis keeps horizontal. These findings suggest that the mechanism of liver failure in cirrhosis, whether compensated and decompensated, could be similar and the same criteria can be used for diagnosis. However, it does not support the view of APASL to exclude decompensated cirrhosis but use the same criteria to diagnose ACLF in compensated cirrhosis and non-cirrhotic chronic liver diseases.

The definition of TB cutoff for liver failure is controversial as well. Though various TB cutoffs have been proposed in relevant studies, they were all initialized from expert consensus or existing models. Actually, few studies illuminated the independent and quantitative correlation between TB level and the short-term mortality of patients. A clinically meaningful cutoff would be one particular value that correlates with a marked change in physiological response and patient outcome (35). Thus, we calculated the TB cutoffs based on the mathematical definition (maximum of acceleration) and the clinical definition (15% 28-day transplantation-free mortality), respectively. In patients with cirrhosis, the two TB cutoffs are 14.2 mg/dL (mathematical) and 18.1 mg/dL (clinical), respectively, both the diagnosis rate (about one fifth) and the corresponding adjusted 28-day mortality (about 15%) were acceptable. In patients with advanced fibrosis, the mathematical TB cutoff is 12.1 mg/dL, whose corresponding mortality (2.9%) was much lower than the clinical definition (15%). In case the TB cutoff of clinical definition (36.0 mg/dL) was applied, only 1.3% patients can be diagnosed as liver failure. Therefore, from the perspective of high short-term mortality, liver failure in advanced fibrosis cannot be diagnosed by TB alone.

This study has several strengths. First, it is the unique study in demonstrating the independent and quantitative correlation between TB and short-term mortality in hospitalized patients with cirrhosis and advanced fibrosis, respectively. Based on the findings of the study, we proved that it is unfeasible to use the same criteria to diagnose liver failure in patients with cirrhosis and advanced fibrosis, settling this protracted controversy between the ACLF consortiums from East and West. Second, the TB cutoffs for liver failure were calculated completely based on mathematical methods, provide evidence-based reference for the establishment of ACLF diagnosis criteria. Third, the study based on the data of hospitalized patients with representative characteristics of cirrhosis and advanced in China, which implied that our findings could be applicable to the 2-billion-population HBV high-epidemic area (15). Finally, the perspective multicenter design will result in low rates of loss to follow-up and missing variables, ensuring the reliability of our conclusions.

Several limitations should be acknowledged. First, we excluded the non-cirrhotic patients with a FIB-4 score <1.45. However, this strategy is implemented to rule-out the patients with merely mild or without fibrosis. The liver conditions of these patients are close to normal, whose features of liver failure (if presented) are similar to that of acute liver failure. Moreover, the 90-day mortality of these patients was only 1.1% (2/178), not the potential victims of ACLF. Second, we excluded the patients received LT within 90 days, which may have some potential inference on the results. However, taking transplant-free mortality as the primary outcome is a common practice in ACLF research to obtain the natural prognosis of patients.

## Conclusion

This study is the first one to clearly demonstrate the different effects of TB on 90-day mortality in patients with cirrhosis and advanced fibrosis, proving that liver failure can be diagnosed by TB alone in cirrhosis but not in advanced fibrosis. We also proposed the first mathematical and clinical TB cutoffs (14.2 and 18.1 mg/dL in cirrhosis, and 12.1 and 36.0 mg/dL in advanced fibrosis) for liver failure via calculation. Our innovative work will become the foundation of the establishing evidence-based ACLF diagnostic criteria in HBV high-endemic areas.

## Data Availability Statement

The original contributions presented in the study are included in the article, further inquiries can be directed to the corresponding author/s at: aclf_group@163.com.

## Ethics Statement

The studies involving human participants were reviewed and approved by the Medical Ethics Board of Shanghai Renji Hospital. The patients/participants provided their written informed consent to participate in this study.

## Author Contributions

HL had full access to all the data used in the study and takes responsibility for the integrity of the data and the accuracy of the data analysis. HL: concept, design, and supervision. LQ and HL: drafting of the manuscript. WZ and HL: critical revision of the manuscript for important intellectual content. LQ and WZ: statistical analysis. HL, GD, XW, XZhe, YHu, JiC, ZM, YG, FL, and XL: obtained funding. All authors: acquisition of data, administrative, technical, or material support.

## Conflict of Interest

The authors declare that the research was conducted in the absence of any commercial or financial relationships that could be construed as a potential conflict of interest.
